# 

**Published:** 1856-10

**Authors:** 


INCLUDING THE

TRANSACTIONS OF THE EPIDEMIOLOGICAL SOCIETY OF LONDON.

EDITED BY

BENJAMIN W. RICHARDSON, M.D.,

PHYSICIAN TO THE ROYAL INFIRMARY FOR DISEASES OF THE CHEST, AND LECTURER ON PUBLIC HYGIENE AT THE GROSVENOR PLACE MEDICAL SCHOOL.

NATIONAL HEALTH IS NATIONAL WEALTH.

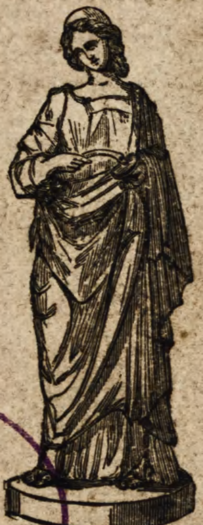

ΥΓΙΕΙΑ
πρεσβίστη μακάρων.

ARMY MEDICAL
OCT 1 - 1946
LIBRARY

LEES-MUSEUM
BIBLIOTHEEK
AMSTERDAM

PRINTED AND PUBLISHED FOR THE PROPRIETOR BY
THOMAS RICHARDS, 37, GREAT QUEEN STREET.
1856.

[Published Quarterly.—Annual Subscription, 10s., Payable in Advance.]

